# Vector Competence of German *Aedes punctor* (Kirby, 1837) for West Nile Virus Lineages 1 and 2

**DOI:** 10.3390/v14122787

**Published:** 2022-12-14

**Authors:** Christin Körsten, Amira A. AL-Hosary, Mandy Schäfer, Birke A. Tews, Doreen Werner, Helge Kampen, Ana Vasic, Cornelia Silaghi

**Affiliations:** 1 Institute of Infectology, Friedrich-Loeffler-Institut, Federal Research Institute for Animal Health, Greifswald-Insel Riems, 17493 Greifswald, Germany; 2 Department of Animal Medicine (Infectious Diseases), Faculty of Veterinary Medicine, Assiut University, Assiut 71526, Egypt; 3 Leibniz-Centre for Agricultural Landscape Research, 15374 Muencheberg, Germany

**Keywords:** *Aedes punctor*, vector competence, West Nile virus, Germany

## Abstract

West Nile virus (WNV) is a zoonotic flavivirus transmitted by mosquitoes as a biological vector. Because of its biting behavior, the widespread snow-melt mosquito *Aedes punctor* could be a potential bridge vector for WNV to humans and nonhuman mammals. However, little is known on its role in transmission of WNV. The aim of this study was to determine the vector competence of German *Ae. punctor* for WNV lineages 1 and 2. Field-collected larvae and pupae were reared to adults and offered infectious blood containing either an Italian WNV lineage 1 or a German WNV lineage 2 strain via cotton stick feeding. Engorged females were incubated for 14/15 or 21 days at 18 °C. After incubation; surviving mosquitoes were dissected and forced to salivate. Mosquito bodies with abdomens, thoraces and heads, legs plus wings and saliva samples were investigated for WNV RNA by RT-qPCR. Altogether, 2/70 (2.86%) and 5/85 (5.88%) mosquito bodies were found infected with WNV lineage 1 or 2, respectively. In two mosquitoes, viral RNA was also detected in legs and wings. No saliva sample contained viral RNA. Based on these results, we conclude that *Ae. punctor* does not play an important role in WNV transmission in Germany.

## 1. Introduction

West Nile virus (WNV, genus *Flavivirus,* family *Flaviviridae*) is one of the most widespread mosquito-borne viruses in the world [1]. To date, at least nine genetic lineages are known, of which lineages 1 and 2 are the most important due to their wide distribution and high virulence [2,3]. WNV circulates in an enzootic cycle between mosquitoes and birds but can also infect several other vertebrate species such as humans and horses [4]. In humans, WNV can remain completely asymptomatic or cause a wide range of symptoms, ranging from flu-like symptoms (West Nile fever, WNF) to severe neurological disease (West Nile neuroinvasive disease, WNND) [4]. Evidence for WNV circulation in Europe was obtained for the first time from Albania in 1958 [5]. Subsequent outbreaks, for instance, in horses in France from 1962 to 1965 [6] or in humans in Romania in 1996 [7], were all associated with WNV lineage 1 [8].

**Figure 1:**
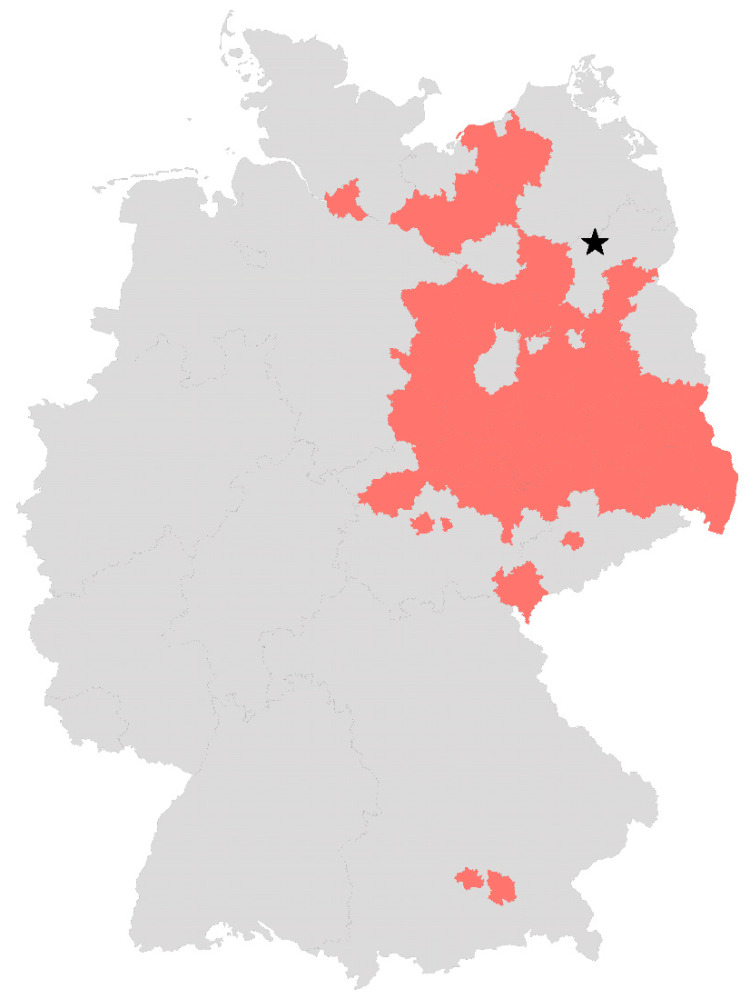
German districts with confirmed WNV infections in birds, horses and humans. Districts that have reported at least one case of WNV infection in birds, horses or humans by 12 November 2022 according to the TierSeuchenInformationsSystem (TSIS) and the European Centre for Disease prevention and control (ECDC) are colored in red. The star marks the collection place of *Ae. punctor* larvae and pupae used in this study. The map was created with Microsoft Excel.

However, in 2004, WNV lineage 2 was found in Europe in a goshawk (*Accipiter gentilis*) in Hungary [9], followed by a wide spread of the lineage in Europe [10,11,12]. Although WNV has been circulating in neighboring countries for many years, it was first diagnosed in Germany in a great grey owl (*Strix nebulosa*) in the federal state of Saxony-Anhalt in August 2018 [13]. In the period from 2019 to 2022, WNV spread in eastern Germany (Figure 1), with only WNV lineage 2 strains being detected [13,14,15]. Numerous autochthonous WNV infections in birds, horses and humans have since been reported, including severe cases of WNF and WNND in humans [15,16,17]. In German mosquitoes, WNV was first found in 2019 in several pools of the *Culex pipiens* complex [18]. The detection of WNV RNA in hibernating *Culex pipiens* biotype *pipiens* females suggest that WNV is able to overwinter in Germany [19].

Vector competence refers to a mosquito species’ ability to acquire, maintain, replicate and transmit a virus [20,21]. It depends on many factors and can even vary within a mosquito species. Various influences such as temperature [22], virus strains [23] or genetic differences between mosquito populations within a species [24] can have an impact on the vector competence.

The main vectors of WNV belong to the *Culex pipiens* complex [25]. However, many other mosquito species also play a role in WNV transmission and maintenance in the natural cycle. In addition to some other *Culex* species (e.g., *Culex modestus* and *Culex torrentium*), several species of the genera *Aedes* and *Anopheles* are also considered as possible vectors, such as *Aedes vexans*, *Aedes cantans*, *Aedes albopictus* and *Anopheles maculipennis* [26,27].

*Aedes (Ae.) punctor* (Kirby, 1837) is a widespread snow-melt mosquito that prefers swampy woodlands as breeding sites and occurs in various European countries, including Germany [28,29,30]. Adults of this univoltine species occur primarily during spring and are mainly mammalo- and anthropophilic (i.e., prefer mammals and humans as hosts) [31,32,33], but also occasionally feed on birds, as shown by field studies and laboratory experiments [32,33,34]. Due to its host range and abundance, *Ae. punctor* might be a potential bridge vector in the transmission cycle of WNV, transmitting the virus from birds to humans and nonhuman mammals [35]. However, neither WNV nor other flaviviruses have yet been detected in field-caught *Ae. punctor* [26,27,36]. In a study by Vermeil et al. [34], *Ae. punctor* females fed on experimentally infected chicks and were able to transmit WNV to native chicks 6 days later; however, further vector competence studies with this species are not known [26,27].

Given the fact that some cases of WNV infections in Germany have been reported in April [37] while *Ae. punctor* is already active, this species might play a role in WNV overwintering and the maintaining and spreading of WNV in Germany. So far, however, it is unknown whether German *Ae. punctor* populations are vector-competent for WNV, and if so, should be considered in WNV surveillance and control.

Therefore, this study aimed to investigate the vector competence of German *Ae. punctor* for WNV lineages 1 and 2 and to determine the need to include this species into risk assessments.

## 2. Materials and Methods

### 2.1. Mosquito Collection, Identification and Rearing

*Aedes punctor* larvae and pupae were collected near Lychen, Germany (Figure 1, N53.177, E13.334) in April 2020 and 2021 and reared to adults under BSL-2 conditions. Species were determined using the identification key by Becker et al. [30]. Larvae, pupae and adults were kept at 24–26 °C, 60–70% relative humidity (RH) and a 16:8 light–dark photocycle. Since adults showed a high mortality, probably due to the temperature exceeding the biological tolerance of this mosquito species in a pretrial, keeping conditions were adjusted to 18 °C with 80% RH and 16:8 light–dark photocycle, resulting in a higher survival rate. Larvae were fed with finely ground TetraMin XL Flakes (Tetra, Melle, Germany), while adults were offered 5% glucose solution ad libitum. A total of 110 adult mosquitoes were randomly chosen for testing for flavivirus negativity, preceding the vector competence trials. These were pooled in groups of 10 specimens, from which nucleic acid was extracted using the QIAGEN RNeasy Mini Kit (Qiagen, Hilden, Germany) according to the manufacturer’s instructions. An RT-qPCR according to Vina-Rodriquez et al. [38] was performed to exclude a previous infection with flaviviruses, which might interfere with the results of the WNV vector competence study.

Confirmation of mosquito species identification was performed using a total of eight randomly selected mosquito body DNA extracts from the infection experiments by amplification and sequencing of the mitochondrial cytochrome c oxidase subunit I gene (COI) [39], using primers according to Folmer et al. [40]. Sequences were compared by BLASTn to records in GenBank.

### 2.2. Cells and Viruses

Mammalian Vero cells received from the Friedrich–Loeffler Institute’s biobank (CCLV-RIE 1146) were used for virus propagation and vector competence studies. WNV lineage 1 (strain Magpie/Italy/203204, GenBank accession no. JF719066) isolated in Italy in 2008 and WNV lineage 2 (strain mosquito/Germany/T-166-60, GenBank accession no. MN921231) isolated in Germany in 2019 were multiplied to titers of 5.62 × 10^7^ and 1.78 × 10^8^ 50% tissue culture infective dose per ml (TCID_50_/mL), respectively.

### 2.3. Vector Competence Study

Vector competence trials were performed in two experiments for each WNV lineage. In 2020, one experiment was performed using WNV lineage 1. In 2021, mosquitoes were used for one repetition with WNV lineage 1 and two infection trial replicates with WNV lineage 2. During all experiments, examination of all or selected mosquitoes was performed 14/15 days after infection. In 2021, some mosquitoes infected with WNV lineages 1 or 2 were incubated for 21 days before processing.

The day before the infection experiments, adult females aged 7–14 days were selected using an oral aspirator with HEPA filter (John W. Hock Company, Gainesville, FL, United States) and placed into transparent plastic tubes with sponge caps (50 mm × 100 mm, Carl Roth, Karlsruhe, Germany) in groups of about 10 specimens and deprived of a sugar source. All infection experiments were performed in a BSL-3 laboratory.

**Figure 2:**
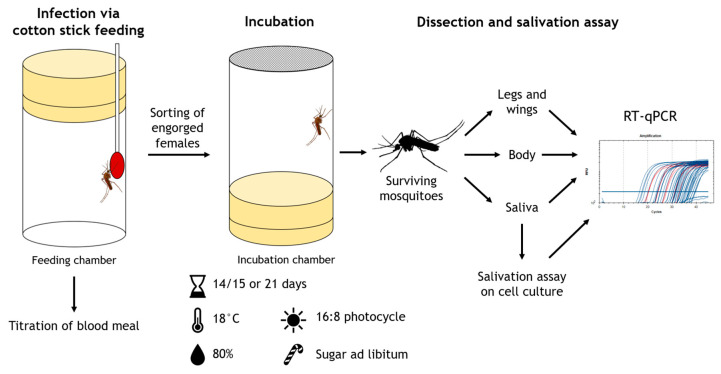
Procedure of vector competence trials.

Procedures of vector competence trials are summarized in Figure 2.

Rabbit blood was used for one infection with WNV lineage 1 in 2020; for all further experiments in 2021, bovine blood was used due to the availability and the need of a higher blood volume. For preparation of an infectious blood meal, virus stocks were mixed in 1:2 ratio with heparinized blood, and 20 µL 5mM ATP (Sigma-Aldrich, St. Louis, MO, USA) per 1ml infectious blood was added. Samples of infectious blood were taken and titrated before and after feeding. Titrations were performed on 1 × 10^5^ Vero cells/mL seeded one day before and incubated for 7 days at 37 °C and 5% CO_2_ in a CO_2_ incubator (Sanyo, Moriguchi, Japan). Cells were fixed and stained with 1% crystal violet solution (Carl Roth, Karlsruhe, Germany) in 7.4% formaldehyde (Carl Roth, Karlsruhe, Germany) for 24 h and then read for CPE using a microscope (Nikon Eclipse Ts2, Nikon Europe B.V., Amstelveen, The Netherlands), to determine blood meal titers.

Cotton sticks were soaked with freshly prepared infectious blood and offered to mosquitoes for 2–3 h. A sample of the infectious blood was kept at room temperature during the feeding and titrated at the end of the feeding. The blood from cotton sticks was not used for titration as this would have been difficult to implement, as well as to avoid bacterial or fungal contamination from the mosquitoes. Engorged females were sorted under CO_2_ anesthesia and placed into incubation chambers (50 mm × 100 mm, Carl Roth, Karlsruhe, Germany) in groups of around 5 to 10 individuals. Incubation chambers were modified plastic tubes whose bottoms were replaced by mesh to allow for feeding and observation of the mosquitoes during incubation. One or two engorged mosquitoes were collected during sorting as control samples on the infection day.

Mosquitoes were incubated for 14/15 or 21 days at 18 °C, 80% RH and 16:8 light–dark photocycle in an incubator (MLR-352H-PE, Panasonic Corporation, Osaka, Japan). During incubation, mosquitoes were offered 5% glucose solution via cotton pads placed on the mesh side of the incubation chambers.

After incubation, surviving mosquitoes were anesthetized with CO_2_ and immobilized by removing their legs and wings. Forced salivation was performed according to Heitmann et al. [41] for 30–45 min, using 20 µL 1× phosphate buffer saline (PBS) per filter tip. By a subsequent addition of further 10 µL 1×PBS, the volume of each saliva sample was increased to 30 µL. In order to detect infectious virus, one part of the saliva sample in 15 µL quantity was immediately placed on a 96-well plate (Corning Incorporated, Corning, NY, USA) seeded with Vero cells one day before. After 45–60 min of incubation, 200 µL minimum essential medium with 2% fetal calf serum, 1% penicillin/streptomycin (Thermo Fisher Scientific, Darmstadt, Germany) and 2% amphotericin B (Sigma-Aldrich, St. Louis, MO, USA) were added to the inoculated wells. Incubation, fixation and staining were performed as described for the blood meal titration. Before staining, cells were checked for cytopathic effect caused by viral growth and 140 µL supernatant of each inoculated well were sampled for later RNA extraction and virus-specific RT-qPCR.

Day 0 controls, mosquito bodies with heads, thoraces and abdomens, legs plus wings, the remaining 15 µL of the saliva sample and the supernatant of the cell culture were placed into 2 mL tubes (Eppendorf, Hamburg, Germany), each filled with 560 µL AVL buffer with carrier RNA (Qiagen, Hilden, Germany). Samples that contained bodies or legs plus wings were additionally macerated (TissueLyser II, Qiagen, Hilden, Germany) for two minutes at 30 Hz using one 3 mm stainless steel bead per tube. All samples were then placed at −80 °C until further analysis.

### 2.4. Nucleic Acid Extractions and Analysis

Prior to nucleic acid extraction, samples were inactivated by incubation at 70 °C for 10 min and centrifuged for 1 min at 13,000 rpm at room temperature (5430R centrifuge, Eppendorf, Hamburg, Germany) [22]. Extraction of RNA and DNA was performed from 200 µL supernatant with the NucleoMag VET Kit (Macherey-Nagel, Düren, Germany) according to the manufacturer’s instructions in a KingFisher Flex (ThermoFisher Scientific, Darmstadt, Germany). To check for success of extraction, 1 µL of an internal control RNA [42] was added to each sample before automatic extraction. A nontemplate negative control was also included during each extraction.

For detection of WNV RNA, a multiplex RT-qPCR was performed with the AgPath-ID One-Step RT-PCR Kit (ThermoFisher Scientific, Darmstadt, Germany), 1 µL primer–probe mix targeting the WNV 5′ nontranslated region (10.0 pmol/µL of each primer and 2.5 pmol/µL FAM-labeled probe) for simultaneous detection of WNV lineage 1 and 2 [43], 1 µL primer–probe mix targeting the internal control RNA (2.5 pmol/µL of each primer and 1.25 pmol/µL HEX-labeled probe) [42] and 5 µL RNA in a total volume of 25 µL. The RT-qPCR was performed in a CFX96 Real-Time PCR system (Bio-Rad Laboratories, Feldkirchen, Germany) under the following conditions: 10 min at 48 °C, 10 min at 95 °C and 45 cycles at 95 °C for 15 s and 60 °C for 45 s. Negative controls from the extractions as well as a nontemplate control of the mastermix were included in each RT-qPCR. Samples with Cq-values of 35–40 in the WNV assay were confirmed by a second PCR assay targeting the WNV NS2A region in both WNV lineage 1 and 2 [43] under the same conditions to exclude false-positive signals. Samples that had a Cq-value of <40 in the second PCR assay were considered positive. Relative quantification was performed based on a standard curve with the Bio-Rad CFX Maestro Software (Bio-Rad Laboratory, Feldkirchen, Germany). For quantification, a 10-fold dilution series of the used virus stocks with known titer was prepared and extracted as described above.

### 2.5. Bloodfeeding, Mosquito Survival and Vector Competence Indices

Feeding rates refer to the total number of engorged females of all living females that were offered infectious blood meal. The survival rate on day 14 indicates the proportion of living mosquitoes of the total number of mosquitoes incubated. Day 0 samples and mosquitoes accidentally killed during sorting were excluded. The survival rate on day 21 refers to the total number of mosquitoes that were not processed at day 14 or 15, regardless of how many were still alive on day 14.

Definition of vector competence indices are based on Vogels et al. [25]. In this study, infection rate (IR) represents the proportion of surviving mosquitoes containing viral RNA in their body, dissemination rate (DR) the proportion of infected mosquitoes with viral RNA in their legs and wings, and transmission rate (TR) the proportion of mosquitoes with disseminated infection containing viral RNA or infectious virus in their saliva. Legs and wings that were found positive in RT-qPCR but whose associated body sample did not contain viral RNA were considered negative.

## 3. Results

### 3.1. Mosquito Species Confirmation amd Flavivirus Screening

To ensure mosquitoes used in the experiments belonged to *Ae. punctor* species, species confirmation was performed with randomly selected mosquito body extracts from the infection experiments by PCR, followed by sequencing and comparison of sequences with GenBank entries. For one body out of eight, only the genus *Aedes* could be confirmed. Since species confirmation was successful for the other seven body samples (99.54–100.00% matches with *Ae. punctor* records from GenBank), the sequencing was not repeated.

In order to exclude any previous flavivirus infection that could interfere with the results of the vector competence experiments, 110 adult mosquitoes were pooled and tested for flavivirus RNA by RT-qPCR. None of the tested pools were found positive.

### 3.2. Titration and Incubation

Two infection experiments were performed with each WNV lineage in order to obtain a number of mosquitoes sufficiently high for the investigations.

Vector competence experiments with WNV lineage 1 were performed in 2020 and 2021. In 2020, rabbit blood was used for oral infection, resulting in a higher feeding rate of 74.65% (53/71) than in 2021, when bovine blood was used and 43.47% (163/375) females fed. Blood meal titers in both infections were 2.37 × 10^6^ and 3.16 × 10^6^ TCID_50_/mL at the beginning of the feeding and 2.37 × 10^5^ and 1.33 × 10^6^ TCID_50_/mL at the end of the feeding, respectively. Engorged females collected as day 0 controls contained WNV RNA of the equivalent of 1.09 × 10^3^, 8.91 × 10^4^ and 1.22 × 10^5^ TCID_50_/mL. Minus the day 0 controls and an accidentally killed mosquito during sorting, a total of 212 engorged females were incubated during both vector competence trials with WNV lineage 1. Fourteen days after infection (dpi), 50.47% (107/212) of the mosquitoes were still alive. Dissection and forced salivation were performed with a total of 58 mosquitoes. Some of the females were further incubated up to 21 dpi. Of the 99 mosquitoes that had been incubated for 21 days, 12.12% (12) were alive and salivated.

Infectious bovine blood containing WNV lineage 2 was offered a total of 372 mosquitoes in 2 infection experiments in 2021. The feeding rates of both trails were comparable with 41.77% (66/158) and 39.25% (84/214). Blood meal titers in both experiments ranged from 2.37 × 10^6^ and 4.22 × 10^6^ TCID_50_/mL at the beginning to 1.33 × 10^5^ and 2.37 × 10^6^ TCID_50_/mL at the end of the feeding, respectively. Day 0 control samples contained WNV RNA in equivalents of 1.81 × 10^5^, 2.11 × 10^5^, 2.53 × 10^5^ and 2.88 × 10^5^ TCID_50_/mL. Minus the day 0 controls, 146 mosquitoes were placed into incubation chambers during both infection experiments, of which 64.38% (94/146) were still alive at 14 dpi. At 14 and 15 days after infection, respectively, 74 mosquitoes were dissected and forced to salivate, while remaining ones were further incubated. At 21 dpi, 50.00% (11) of the 22 mosquitoes in the remaining chambers were still alive and processed.

### 3.3. Infection

In total, 15 samples containing legs and wings were found to be positive in RT-qPCR, while the associated body sample as well as the saliva samples did not contain viral RNA. Consequently, these samples were assumed to be negative. A contamination during sample processing was ruled out, as all negative controls from extraction and RT-qPCR were found negative for WNV RNA. In order to rule out potential false positive signals in other samples, a second WNV assay was performed as described above.

Results of the vector competence experiments are shown in Table 1.

Results of RT-qPCR and relative quantification of all positive samples can be found in Table 2.

The results of the day 0 samples examined by RT-qPCR showed that engorged females who were incubated had taken up the virus during all infection experiments.

Overall, the bodies of only 2.86% (2/70) of the mosquitoes were found to be infected with WNV lineage 1. Infected mosquito bodies contained viral RNA equivalent to 2.61 × 10^3^ and 8.38 × 10^3^ TCID_50_/mL, respectively. One of these mosquitoes had a disseminated infection (1/2, 50.00%). Both infected mosquitoes were processed at 14 dpi. Neither viral RNA nor evidence of infectious virus was found in any of the saliva samples.

A total of 5.88% (5/85) of the mosquito bodies were found infected with WNV lineage 2. Four of them were processed at 14 dpi and one at 21 dpi. The amount of viral RNA in mosquito bodies ranged in equivalent between 7.71 × 10^2^ and 1.29 × 10^8^ TCID_50_/mL. One mosquito processed at 14dpi had a disseminated infection (1/5, 20.00%). No evidence of viral presence was found in the saliva samples.

The low infection rates and the absence of viral RNA in the saliva samples during all infection experiments with both WNV lineages indicate a low susceptibility and vector competence of *Ae. punctor* for the tested WNV strains.

## 4. Discussion

The snow-melt mosquito *Ae. punctor* (Kirby, 1837) could be a potential bridge vector for WNV transmission from birds to humans because of its distribution and biting behavior [35]. In Germany, this species might play a role in the maintenance and spreading of the virus. So far, however, there have been no studies examining the vector competence of German *Ae. punctor* and the resulting consequences for WNV surveillance and control in Germany.

In our study, *Ae. punctor* showed a low susceptibility to WNV infection. Due to the low infection rates, it is difficult to make a precise statement about the rates of dissemination and transmission in this mosquito species. Nevertheless, in 70 and 85 mosquitoes examined for WNV lineages 1 or 2, respectively, no WNV RNA was found in saliva samples, suggesting a low vector competence of this species for both WNV strains tested.

Up to now, *Ae. punctor* has rarely been investigated in vector competence studies [27]. However, in a study by Vermeil et al. [34], *Ae. punctor* that fed on experimentally infected chicks were able to transmit WNV to noninfected chicks after incubation at 25 °C for six days. Since only ten mosquitoes were investigated, a significantly higher infection rate than in our study can be assumed.

In order to infect a mosquito and to be transmitted during a mosquito’s bite, arboviruses such as WNV have to overcome several barriers in the mosquito vector [21,44]. After being taken up with the blood meal, the arbovirus must first infect the mosquito midgut cells, which is the most important barrier determining the vector competence [45,46]. Reasons for a strong midgut infection barrier could be the lack of receptors used by WNV, or an efficient immune response through, for example, the RNA interference pathway [20,46,47,48]. Due to the low infection rates observed in our study, the midgut barrier of the investigated German *Ae. punctor* appears to be the limiting factor in their vector competence. This is in contrast to the higher susceptibility of the *Ae. punctor* population investigated by Vermeil et al. [34]. One possible explanation might be a variation of susceptibility to the different WNV strains used. While our WNV strains were both isolated in Europe, Vermeil et al. [34] used an African WNV strain. A genetic difference between the viral strains could explain the variation in the infection rates [21,23]. However, in our study, there was no difference in susceptibility of the mosquitoes to the genetically distinct WNV lineages 1 and 2. Therefore, a genetic difference between the tested mosquito strains is more likely. Vector competence varies between distinct mosquito species, but might also differ between populations of the same species [24,49], and the *Ae. punctor* populations investigated by Vermeil et al. [34] and in the present study have not only a geographically different origin, but also a temporally different one.

Susceptibility to a virus is also dependent on the dose of ingested virus. A higher WNV titer in a blood meal can result in an increased infection rate [50]. Vermeil et al. [34] used inoculated viremic chicks as a source of the infectious blood meal and indicated a blood titer of 1 × 10^4^ to 1 × 10^6^ 50% infective dose per ml. In our study, blood meal titers ranged between 1.33 × 10^5^ to 4.22 × 10^6^ TCID_50_/mL, and should therefore have been sufficient to cause an infection in *Ae. punctor.* Other studies also showed that the viremia that can be expected in WNV-infected chickens is not higher than the blood meal titers that we used [51,52]. In addition, the amount of ingested viral RNA detected in day 0 controls was not lower than in other vector competence studies where mosquito infection was successful [49,53]. Thus, the blood meal titer does not appear to be the reason of the low positivity rates. However, the differences in blood feeding methods might have an impact on the susceptibility of *Ae. punctor*. It has been shown that the presence of the flavivirus nonstructural protein 1 in sera of viremic hosts can enhance acquisition of virus in mosquitoes [54]. Therefore, the use of infected animals as a source of infectious blood might have led to higher positivity rates compared to our artificial produced infectious blood.

Another factor affecting vector competence studies is the incubation temperature, as higher temperatures can enhance WNV replication and therefore increase infection and transmission rates in mosquito vectors [23,55,56]. All incubations in our study were performed at 18 °C. The choice of 18 °C as the incubation temperature was based on the observed higher survival of *Ae. punctor* at 18 °C compared to the initial incubation at 24–26 °C. In addition, an incubation temperature of 18 °C represents the average spring temperature in Germany (average temperature in May 2021 was 10.7 °C) [57], which is much better than higher temperatures of 23–28 °C that are often used in vector competence trials with WNV [25]. Vermeil et al. [34] used a temperature of 25 °C, which may have increased the infection rates in *Ae. punctor.* It is conceivable that a higher incubation temperature would have led to higher positivity rates in our *Ae. punctor* as well. However, how efficiently a vector species transmits a specific virus does not only depend on its vector competence, but on several other factors such as abundance and daily survival of the mosquito and is summed up as vector capacity [58]. The *Ae. punctor* mosquitoes used in our study showed a high mortality when reared at 24–26 °C. Even with more efficient development of WNV due to higher temperatures, it can be assumed that the simultaneously increased mortality would still result in a low vector capacity of *Ae. punctor*. However, it remains questionable whether the low incubation temperature alone is responsible for the low susceptibility observed in our study. Vermeil et al. [34] were able to detect WNV transmission by *Ae. punctor* already six days after infection. In our study, mosquitoes had a longer incubation period of 14/15 or 21 days, respectively. Even after three weeks, we neither observed higher infection rates nor transmission. Although other studies found reduced WNV susceptibility at lower temperatures, *Cx. pipiens* mosquitoes were still able to transmit WNV when incubated at 18 °C for 14/15 and 20/21 days [22,55], indicating that temperature-dependent slower replication might not be the only reason for the low susceptibility of *Ae. punctor* for WNV in our study.

In field-collected European *Ae. punctor,* several other arboviruses were detected. Almost all of them belong to the genus *Orthobunyavirus* (order *Bunyavirales*), such as Batai virus [59], Inkoo virus [60] and Tahyna virus [61,62]. Furthermore, *Ae. punctor* is a known vector of the orthobunyaviruses Jamestown Canyon virus [30] and Snowshoe hare virus [63,64]. In addition to the orthobunyaviruses, Sindbis virus (genus *Alphavirus*, family *Togaviridae*) was detected in *Ae. punctor* larvae in Sweden [65]. Despite all these detections of arboviruses, there is no record to this date of mosquito-borne flaviviruses in field-collected *Ae. punctor* [26,36,62]. It therefore appears that *Ae. punctor* might not be suitable vectors for flaviviruses at all. Since the vector competence of this species was experimentally proven [34], extrinsic influences such as temperature appear to be the limiting factors.

## 5. Conclusions

German *Ae. punctor* showed low infection rates and no evidence of transmission after experimental infection with WNV lineage 1 (strain Magpie/Italy/203204) and WNV lineage 2 (strain mosquito/Germany/T-166-60) in this study. We therefore conclude that this mosquito species does not play a role in WNV transmission and does not need to be targeted by future surveillance and control programs.

## Figures and Tables

**Table 1:** Infection (IR), dissemination (DR) and transmission rates (TR) of *Ae. punctor* for WNV lineages 1 (strain Magpie/Italy/203204) and 2 (strain mosquito/Germany/T-166-60).

Virus	Incubation Period	IR *n*/*n* (%)	DR *n*/*n* (%)	TR *n*/*n* (%)
WNV lineage 1	14 days 21 days	2/58 (3.45) 0/12 (0.00)	1/2 (50.00) 0/0 (0.00)	0/0 (0.00) 0/0 (0.00)
WNV lineage 2	14/15 days 21 days	4/74 (5.41) 1/11 (9.09)	1/4 (25.00) 0/0 (0.00)	0/0 (0.00) 0/0 (0.00)

**Table 2:** Results of quantification by RT-qPCR of the viral load in mosquito bodies, legs and wings of *Ae. punctor* infected with WNV lineages 1 (strain Magpie/Italy/203204) and 2 (strain mosquito/Germany/T-166-60).

Virus	Sample	Incubation Period (Days)	Bodies		Legs and Wings	
			Cq-Value	Corresponds to TCID_50_/mL ^1^	Cq-Value	Corresponds to TCID_50_/mL ^1^
WNV lineage 1	#1 #2	14 14	32.80 32.00	8.38 × 10^3^ 2.61 × 10^3^	ND 33.57	ND 1.08 × 10^3^
WNV lineage 2	#1 #2 #3 #4 #5	14 14 15 15 21	17.71 34.91 21.98 37.95 37.94	1.29 × 10^8^ 1.21 × 10^3^ 2.66 × 10^7^ 7.71 × 10^2^ 5.52 × 10^2^	33.11 ND ND ND ND	4.09 × 10^3^ ND ND ND ND

^1^ titer equivalent to the amount of WNV RNA; ND, not detected.

## Data Availability

Not applicable.
